# A registry-based follow-up study, comparing the incidence of cardiovascular disease in native Danes and immigrants born in Turkey, Pakistan and the former Yugoslavia: do social inequalities play a role?

**DOI:** 10.1186/1471-2458-11-662

**Published:** 2011-08-23

**Authors:** Nana F Hempler, Finn B Larsen, Signe S Nielsen, Finn Diderichsen, Anne H Andreasen, Torben Jørgensen

**Affiliations:** 1 Research Centre for Prevention and Health, the Capital Region, Glostrup University Hospital, Building 84/85, 2600 Glostrup, Denmark; 2 Centre of Public Health, Central Denmark Region, 8200 Aarhus N, Denmark; 3 Danish Research Centre for Migration, Ethnicity and Health, Section for Health Services Research, Department of Public Health, University of Copenhagen, 1014 Copenhagen K, Denmark; 4 Section for Social Medicine, Department of Public Health, University of Copenhagen, 1014 Copenhagen K, Denmark

## Abstract

**Background:**

This study compared the incidence of cardiovascular disease (CVD) and acute myocardial infarction (AMI) between native Danes and immigrants born in Turkey, Pakistan and the former Yugoslavia. Furthermore, we examined whether different indicators of socioeconomic status (SES), such as employment, income and housing conditions influenced potential differences.

**Methods:**

In this registry-based follow-up study individuals were identified in a large database that included individuals from two major regions in Denmark, corresponding to about 60% of the Danish population. Incident cases of CVD and AMI included fatal and non-fatal events and were taken from registries. Using Cox regression models, we estimated incidence rates at 5-year follow-up.

**Results:**

Immigrant men and women from Turkey and Pakistan had an increased incidence of CVD, compared with native Danish men. In the case of AMI, a similar pattern was observed; however, differences were more pronounced. Pakistanis and Turks with a shorter duration of residence had a lower incidence, compared with those of a longer residence. Generally, no notable differences were observed between former Yugoslavians and native Danes. In men, differences in CVD and AMI were reduced after adjustment for SES, in particular, among Turks regarding CVD. In women, effects were particularly reduced among Yugoslavians in the case of CVD and in Turks in the case of CVD and AMI after adjustment for SES.

**Conclusions:**

In conclusion, country of birth-related differences in the incidence of CVD and AMI were observed. At least some of the differences that we uncovered were results of a socioeconomic effect. Duration of residence also played a certain role. Future studies should collect and test different indicators of SES in studies of CVD among immigrants.

## Background

According to a European collaborative project, reliable and comparable data on cardiovascular disease (CVD) in migrant and ethnic minority groups are scattered, incomplete and missing in most European countries [1]. Some European studies have reported marked differences in cardiovascular disease by country of birth and ethnicity in terms of CVD mortality [2-6] and in the incidence of CVD and subcategories of CVD [7-15].

In Denmark, it is challenging to conduct epidemiological studies concerning immigrants' health, because Denmark is a small country and has one of the smallest immigrant populations in Western Europe. Furthermore, immigrants and, in particular, their descendants comprise a very young population due to a relatively short history of immigration. In 2010, immigrants and their descendants constituted about 10% of the total population, and two thirds originated from a non-Western country [16]. Labour migrants settled in Denmark in the late 1960s due to the economic boom, particularly immigrants born in Turkey, Pakistan and the now former Socialist Federal Republic of Yugoslavia (former Yugoslavia). Currently, the abovementioned immigrant groups represent the groups with the longest duration of residence and the highest average age in Denmark from non-Western countries [17].

So far, no Danish follow-up study has investigated the incidence of CVD in relation to country of birth. Current data on CVD in immigrants in Denmark is conflicting. National statistics has reported a decreased mortality among immigrants from non-Western countries, compared with native Danes [17]. On the contrary, a registry-based report, published by the Danish National Board of Health, has reported an increased use of hospital services related to CVD among immigrant groups from non-Western countries, compared with native Danes [18].

Another aspect of great importance is the well documented association between socioeconomic status (SES) and CVD. In several Western countries, individuals of low SES have higher rates of CVD morbidity and mortality than individuals of high SES [19]. Non-Western immigrants in Denmark are often disadvantaged regarding socioeconomic conditions in terms of income, employment and educational level, compared with native Danes [17]. Furthermore, previous studies have demonstrated that the association between SES and several health outcomes differs between immigrant groups, compared to native populations [20-22]. However, there is a lack of clarity about to which extent and how poor socioeconomic status influences the health of immigrants. The relationship between the SES measure and the outcome measure might differ between immigrants and the native population, suggesting an interaction between SES and country of birth, in relation to health outcomes. However, low SES can also be perceived as a consequence of immigration, caused by poorer access to job opportunities, poorer living conditions e.g. that eventually will influence immigrants' health.

Being able to efficiently prevent, diagnose and treat cardiovascular disease among immigrants, knowledge is needed about the incidence of cardiovascular diseases. In this study, we investigated the incidence of CVD according to country of birth. We also examined the incidence of AMI, as it normally leads to hospitalization and therefore reduces the risk of selection bias caused by differences in seeking primary care and hospital care between immigrants and native Danes. Furthermore, we hypothesized that potential differences between immigrants and native Danes were influenced by socioeconomic factors. Therefore, we examined whether an effect modification between different indicators of SES and country of birth existed, or whether SES indicators were mediators of the effect of country of birth on the incidence of CVD and AMI.

## Methods

### Study population

We undertook a registry-based follow-up study with a population that consisted of Native Danes and immigrants born in Turkey, Pakistan and the former Yugoslavia. Individuals were identified in a database comprising individuals that lived in the Capital Region or Central Denmark Region of Denmark on 1 January 2001 (n = 3,107,901). The total Danish population comprises around 5,500,000 inhabitants. We included individuals that were above 29 years of age with residence in Denmark between 1997 and 2000 (n = 1,837,707); immigrants constituted 26,341. The population was identified in the Central Population Registry in which all residents in Denmark are registered by a unique 10 digital number, making linkage across time and registries possible. The data used from the Central Population Registry is openly available.

### Outcome data

Data on incident cases of CVD and AMI included hospital contacts and deaths in and outside of hospital. Diagnoses are not registered by the general practitioners in Denmark and are therefore not included in our study. Data were obtained from the Registry of Causes of Death and the Danish National Patient Registry, using the International Classification of Diseases ICD-10. CVD codes (I11, I13, I20-I25, I44-I50, I60-I69 (excluding I67.1, I67.5, I67.7, I68), I70-I73) and AMI codes (I21, I22). Individuals with a previous history of CVD (N = 109,769) or AMI (N = 15,834) between 1997 and 2000 were excluded, leaving 1,727,938 individuals for analyses of CVD and 1,821,873 for analyses of AMI. Incident cases included admissions with primary diagnosis or death as underlying or contributing cause of death. Information on deaths and migrations was obtained through linkage to the Registry for Population Statistics at Statistics Denmark. Person-years were calculated from 1 January 2001 to first incident case of CVD and AMI. Individuals were censored when they died, emigrated or at the end of the follow-up period in 2005.

### Covariates

From the Central Population Registry, the Demographic Database and the Integrated Database for Labour Marked Research (IDA) at Statistics Denmark information on country of birth, age, sex, duration of residence, marital status, income, employment and housing conditions in 2001 were obtained.

Analyses were stratified by sex and were age-adjusted. Age was included as a continuous variable. Immigrants comprised individuals born in a foreign country to parents without Danish citizenship also born in a foreign country. Individuals from Yugoslavia did not include refugees that arrived to Denmark in the 1990s, due to the Yugoslavian civil war, because of a change in the recording practice. Native Danes comprised individuals with at least one parent, who was a Danish citizen born in Denmark. Marital status was defined as single (living alone or widow) and living with a partner (married or cohabitation).

Socioeconomic data were based on data from the preceding year. Data on annual income were wages from earnings and social transfers by tax authorities (using currency rate 100 DKK = 13.42 EUR). In individuals living with a partner, the average income in both was calculated. Income was included as a continuous variable. Employment was defined as: employed, out of work or retired. Housing conditions included two categories: self-owned housing and rental-occupied housing. Information on education was not included, due to poor validity. First, for a considerable number of immigrants, particularly women, data did not contain information on achieved educational level in country of birth. Secondly, the variable contained data from a questionnaire survey conducted by Statistics Denmark in 1999 among immigrants, which leaves uncertainty about the variable due to potential selection bias and validity of answers.

### Statistical analysis

Chi-square test or wilcoxon test was used for univariate analyses. Cox proportional hazard regression models were carried out to analyse incidence rates of CVD and AMI. As model control, we tested the assumptions of proportional hazards with Schoenfeld residuals. For both models, assumptions were fulfilled. Linearity of age and income were tested, and when needed we squared the number, or raised it to the third power. We tested plausible interaction terms between country of birth and the socioeconomic indicators. In case of no significant interaction terms between country of birth and SES indicators, we included SES as intermediate variables. Socioeconomic indicators were added stepwise in the models based on assumptions about causal relations, e.g. employment was included prior to income, because employment is assumed to affect income, and furthermore income was included prior to housing, because income is assumed to affect housing. We also carried out separate analyses with each of the SES indicators. Interaction terms, between marital status and country of birth and duration of residence and country of birth, were also tested. In these models, SES indicators and age were included. The analyses, of the impact of duration of residence, were performed within immigrant groups and also with native Danes as the reference category. We stratified the analyses by 15 years age groups (30-44 years and 45-60 years), because age and duration of stay were closely related. Furthermore, these analyses were performed only for CVD and for men and women combined, due to low cell count. Data were analysed using SAS statistical software version 9.1 (SAS Institute Inc., Cary, NC, USA).

## Results

Men were slightly overrepresented in immigrants groups, whereas the opposite was the case in native Danes (Table 1). Native Danes were more likely to be older, compared with the immigrant groups. Immigrant groups were more likely to be living with a partner, be out of work and less likely to be retired, compared with native Danes. Annual income was lower among all immigrants groups, compared with native Danes. Furthermore, immigrants were more likely to live in rental-occupied housing.

**Table 1:** Baseline characteristics (2001) according to country of birth

	Denmark (n = 1 811 366)		Turkey (n = 13 379)				Former Yugoslavia (n = 6 936)				Pakistan (n = 6 026)			
	**Men**	**Women**	**Men**		**Women**		**Men**		**Women**		**Men**		**Women**	
	48.1%	51.9%	51.1%***		48.9%***		51.6%***		48.4%***		55.8%***		44.2%***	

**Age in years**														
Mean	52.2	54.8	43.3***		42.6***		46.6***		47.5***		46.5***		44.6***	

**Marital status**														
Partner	61.2%	54.8%	89.8%***		86.3%***		75.8%***		68.5%***		89.5%***		86.6%***	
Single	38.8%	45.2%	10.2%		13.7%		24.2%		31.5%		10.5%		13.4%	

**Duration of residence (years)**														
> 20			61.1%		50.2%		59.0%		59.5%		65.6%		57.3%	
5-20			38.9%		49.8%		41.0%		40.5%		34.4%		42.7%	

**Income (EUR)**														
Mean	21 155	19 509	11 648***		11 115***		14 068***		13 055***		10 773***		10 427***	

**Employment**														
Employed	67.1%	55.6%	59.7%***		39.4%***		57.7%***		43.3%***		60.2%***		29.4%***	
Out of work	11.1%	14.3%	29.3%		53.1%		30.1%		44.7%		27.9%		64.4%	
Pensioners	21.8%	30.1%	10.9%		7.4%		12.1%		11.9%		11.9%		6.2%	
Missing	0.0%	0.0%	0.1%		0.1%		0.1%		0.1%		0.0%		0.0%	

**Housing conditions**														
Self-owned	3.2%	57.5%	11.7%***		10.2%***		22.3%***		21.9%***		22.8%***		20.0%***	
Rental-occupied	35.6%	41.7%	86.9%		89.4%		76.1%		77.2%		75.6%		79.5%	
Missing	1.2%	0.8%	1.4%		0.4%		1.6%		0.9%		1.6%		0.5%	

**AMI^a^**														
Incident cases^c^	19 233	12 832	125		31		42		20		90		28	
Person-years	4 124 597	4 500 544	35 101		30 548		17 191		16 515		15 426		12 910	

**CVD^b^**														
Incident cases^c^	64 558	60 390	333		173		161		129		199		102	
Person-years	3 833 377	4 243 450	33 390		29 211		16 186		15 578		14 371		12 378	

Chi-square test or wilcoxon test (reference group: native Danish women and men) *** P-value < 0.0001

a: Individuals with previous history of AMI between 1997 and 2000 were excluded

b: Individuals with previous history of CVD between 1997 and 2000 were excluded

c: Including fatal and non-fatal cases

### Interaction terms of marital status and SES

No significant interaction terms were found between marital status and country of birth. Furthermore, no significant interaction terms were found between country of birth and SES indicators in relation to AMI or CVD. P-values for AMI were all above 10%, but because some p-values for CVD were close to a 5% significance level, analyses for CVD were stratified by SES (Table 2). The stratified analyses showed a relatively consistent pattern among women regarding the influence of SES, with the exception of employment, in which retired Pakistani and Turkish women had a lower incidence, compared with those employed, while the opposite was the case among native Danes and Former Yugoslavian women (Table 2). In men, we found a similar pattern of the influence of income across the groups, with the exception of Turkish men. Regarding employment, we observed that immigrant groups that were retired had a lower incidence of CVD, compared to those employed, whereas retired native Danes had an increased incidence. Regarding the influence of housing conditions, no differences were observed between homeowners and non-homeowners among immigrant men, dissimilar to native Danish men, in which non-homeowners had an increased incidence.

**Table 2:** Stratified analyses of SES in relation to incidence of CVD according to country of birth

	Denmark	Turkey	Former Yugoslavia	Pakistan
**Men **				
Employment (P = 0.1065)*				
Employed	1	1	1	1
Out of work	**1.82 (1.77-1.87) **	**1.36 (1.03-1.80) **	1.06 (0.72-1.55)	**1.64 (1.17-2.29) **
Retired	**1.15 (1.12-1.18) **	**0.63 (0.43-0.94) **	**0.53 (0.30-0.91) **	0.93 (0.59-1.46)
Income (P = 0.0886) *				
High (above average)	1	1	1	1
Low (below average)	**1.36 (1.33-1.38) **	0.79 (0.51-1.21)	1.25 (0.70-1.98)	1.25 (0.68-2.31)
Housing (P = 0.0712)*				
Self-owned	1	1	1	1
Rental-occupied	**1.55 (1.23-1.28) **	1.00 (0.71-1.38)	1.07 (0.80-1.50)	1.05 (0.63-1.40)
				
**Women **				
Employment (P = 0.0611)*				
Employed	1	1	1	**1 **
Out of work	**1.90 (1.90-2.03) **	1.11 (0.70-1.76)	1.80 (0.99-2.30)	**1.67 (1.06-1.65) **
Retired	**1.37 (1.32-1.42) **	0.65 (0.33-1.28)	1.12 (0.46-2.72)	0.46 (0.23-0.95)
Income (P = 0.4811)*				
High (above average)	**1**	1	1	1
Low (below average)	**1.46 (1.42-1.49) **	1.81 (0.79-4.18)	1.26 (0.75-2.10)	1.32 (0.57-3.05)
Housing conditions (P = 0.3115)*				
Self-owned	**1**	1	**1**	1
Rental-occupied	**1.25 (1.23-1.28) **	0.93 (0.57-1.51)	**1.73 (1.08-2.77) **	1.46 (0.87-2.47)

The values represent Hazard ratio estimates (95% confidence interval)

All models are adjusted for marital status and age

*P-values from Cox regression analyses (country of birth*SES)

### CVD incidence

Turkish and Pakistani men had an increased incidence of CVD, compared with native Danes (Table 3, model 1). After adjustment for employment, income and housing estimates were reduced, but remained significant in Pakistani men. Yugoslavian men had a lower incidence of CVD after adjustment for SES. In women, all immigrant groups had an increased incidence of CVD, compared with native Danish women (Model 1). After adjustment for SES, estimates were no longer significant in Yugoslavian and Turkish women, whereas in Pakistani women, estimates attenuated but remained significant. Employment was the SES indicator that reduced differences the most, in particular among women.

**Table 3:** Incidence of CVD according to country of birth

	Turkey	Former Yugoslavia	Pakistan
**Men**			
Model 1	**1.35 (1.21-1.50) **	1.00 (0.86-1.17)	**1.51 (1.32-1.75) **
Model 2	**1.20 (1.08-1.34) **	0.89 (0.76-1.03)	**1.37 (1.19-1.57) **
Model 3	**1.17 (1.05-1.31) **	0.87 (0.74-1.03)	**1.33 (1.16-1.53) **
	1.24 (1.12-1.39)*	0.93 (0.79-1.08)*	1.40 (1.22-1.61)*
Model 4	1.09 (0.97-1.22)	**0.83 (0.71-0.97) **	**1.26 (1.10-1.45) **
	1.20 (1.08-1.34)**	0.92 (0.79-1.08)**	1.40 (1.22-2.15)**
			
**Women **			
Model 1	**1.52 (1.21-1.77) **	**1.36 (1.14-1.64) **	**1.90 (1.56-2.31) **
Model 2	**1.21 (1.04-1.41) **	1.14 (0.95-1.39)	**1.46 (1.20-1.77) **
Model 3	**1.19 (1.03-1.39) **	1.14 (0.96-1.36)	**1.43 (1.17-1.74) **
	1.44 (1.24-1.67)*	**1.28 (1.88-1.52**)*	1.77 (1.46-2.15)*
Model 4	1.11 (0.95-1.29)	1.10 (0.93-1.31)	**1.35 (1.11-1.64) **
	1.39 (1.19-1.61)**	1.28 (1.08-1.52)**	1.77 (1.45-2.15)**

The values represent Hazard ratio estimates (95% confidence interval)

Reference group: native Danes

Model 1: adjusted for marital status and age

Model 2: adjusted for employment, marital status and age

Model 3: adjusted for income, employment, marital status and age

Model 4: adjusted for housing conditions, income, employment, marital status and age

*employment not included in model 3

**employment and income are not included in model 4

### AMI incidence

With respect to AMI, immigrant men from Turkey and Pakistan had an increased incidence, compared with native Danish men, whereas Yugoslavian men did not differ from native Danish men (Table 4, model 1). When adjusting the models for employment, income and housing, estimates attenuated, but remained significant. Women from Pakistan and Turkey had an increased incidence of AMI, and estimates were reduced after adjustment for SES, but remained significant in Pakistani women. Yugoslavian women did not differ from Danish women. After adjustment for SES, an insignificant trend of lower incidence was observed among Yugoslavian men and women. In addition, employment among women, as a single SES indicator, had a stronger effect compared with income and housing.

**Table 4:** Incidence of AMI according to country of birth

	Turkey	Former Yugoslavia	Pakistan
**Men**			
Model 1	**1.74 (1.45-2.08) **	0.89 (0.66-1.21)	**2.32 (1.88-2.85) **
Model 2	**1.57 (1.31-1.87) **	0.81 (0.60-1.10)	**2.13 (1.73-2.62) **
Model 3	**1.45 (1.21-1.73) **	0.76 (0.56-1.04)	**1.95 (1.58-2.40) **
	1.52 (1.27-1.81)*	0.81 (0.60-1.10)*	2.04 (1.60-2.52)*
Model 4	**1.36 (1.14-1.63) **	0.75 (0.55-1.01)	**1.89 (1.54-2.33) **
	1.54 (1.29-1.85)**	0.84 (0.62-1.14)**	2.15 (1.75-2.65)**
			
**Women **			
Model 1	**1.48 (1.04-2.11) **	1.06 (0.68-1.64)	**3.05 (2.10-4.42) **
Model 2	1.16 (0.81-1.65)	0.91 (0.59-1.42)	**2.26 (1.56-3.28) **
Model 3	1.12 (0.79-1.59)	0.87 (0.56-1.35)	**2.18 (1.50-3.16) **
	1.38 (0.97-1.96)*	0.98 (0.63-1.53)*	2.79 (1.92-4.04)*
Model 4	1.05 (0.74-1.50)	0.80 (0.51-1.26)	**2.08 (1.43-3.02) **
	1.37 (0.96-1.95)**	0.95 (0.61-1.50)**	2.86 (1.97-4.14)**

The values represent Hazard ratio estimates (95% confidence interval)

Reference group: native Danes

Model 1: adjusted for marital status and age

Model 2: adjusted for employment, marital status and age

Model 3: adjusted for income, employment, marital status and age

Model 4: adjusted for housing conditions, income, employment, marital status and age

*employment not included in model 3

**employment and income not included in model 4

### Duration of residence and incidence of CVD

Regarding duration of residence, significant interaction terms were observed (age group 30-44, p = 0.0255 and age group 45-60, p < 0.001), and as a result, analyses were stratified by duration of residence (Table 5). Turks and Pakistanis with a short duration of residence had a lower incidence of CVD, compared with those with a long duration of residence, but only significant among Pakistanis (age group 30-44) and Turks (age group 45-60). Among former Yugoslavians the reverse pattern was observed; though, estimates were not significant and confidence intervals were broad. Using native Danes as the reference category, results were more inconsistent; however, we observed a tendency of high CVD incidence among immigrants with a long duration of residence.

**Table 5:** Incidence of CVD and duration of residence

Duration of residence (years)	Turkey*	Former Yugoslavia*	Pakistan *
Incident cases (30-44 years)/person-years	89/42 180	33/16 094	38/13 909
Incident cases (45-60) years)/person-years	214/15 283	131/10 946	169/10 195

Age group 30-44 years			
5-20	0.64 (0.41-1.00)	1.35 (0.63-2.91)	**0.39 (0.20-0.77) **
> 20 (ref)	1	1	1

Age group 45-60 years			
5-20	**0.53 (0.35-0.78) **	1.24 (0.80-1.92)	0.83 (0.53-1.31)
> 20 (ref)	1	1	1

Age group 30-44 years**			
5-20	0.77 (0.38-1.54)	1.16 (0.58-2.30)	0.92 (0.41-2.06)
> 20	1.12 (0.85-1.47)	0.76 (0.41-1.41)	**1.66 (1.11-2.49) **

Age group 45-60 years**			
5-20	0.63 (0.35-1.11)	1.15 (0.67-2.00)	1.08 (0.59-1.98)
> 20	**1.26 (1.09-1.46) **	1.02 (0.84-1.24)	**1.40 (1.19-1.65) **

The values represent Hazard ratio estimates (95% confidence interval)

*Adjusted for marital status, age, sex, employment, income and housing conditions

** Reference group: Native Danes

### Additional analyses

We repeated analyses, where we restricted the study population to individuals below 65 years of age, because associations between disease and SES are the least in the elderly. A similar pattern for CVD and AMI according to country of birth was observed; however, women from Turkey did not differ from native Danish women in relation to AMI (results not shown), but findings were the same, when we adjusted for SES. Furthermore, because there were cases among those below the age of 30 years, we repeated the analyses (Model 1), by including individuals above 18 years of age; however, the estimates did not change notably in the case of AMI or CVD (results not shown).

## Discussion

The main findings were that immigrants from Turkey and Pakistan had an increased incidence of CVD and AMI, whereas former Yugoslavians did not differ markedly from native Danes. After adjustment for SES, estimates were reduced, in particular, among Turkish men in the case of CVD, in Yugoslavian women in the case of CVD, and in Turkish women in the case of AMI and CVD. The findings of our study are in line with the few European follow-up studies comparing incidence of CVD among immigrants and non-immigrants. Hedlund et *al*. also found an increased incidence of myocardial infarction among Turkish immigrants, whereas no differences were observed between native Swedes and former Yugoslavians [9]. Furthermore, other Swedish studies have reported an increased incidence of CVD and coronary heart disease among Turkish immigrants [7,15]. However, studies have had divagating findings regarding the influence of SES on the incidence of CVD according to country of birth. One study reported that the increased incidence of myocardial infarction among foreign-born individuals was not influenced by socio-economic differences [9], while other studies have reported some or little effect of SES in relation to the incidence of stroke, CVD and CHD [7,14]. Variations might be a result of differences in the design of the studies, including the definition of incident cases and SES. The particular SES factor applied to reflect SES, and when and how it is measured, including the validity, might affect conclusions. In addition, similar immigrant groups might not be comparable across countries, due to differences in migration histories and integration policies in the receiving countries.

As previously mentioned, we decided to include stratified analyses of SES for CVD by country of birth, in order to investigate possible patterns. For some SES indicators, we found similar patterns across country of birth, but in other cases, particularly among men in terms of housing and employment, we found differences in how SES influenced the incidence of CVD. Although the stratified analyses suggested that the relationship between SES indicators and CVD in some cases differed by country of birth, the analyses should be interpreted with caution, due to a lack of significant interaction terms between SES and country of birth and the relatively small sample size among immigrants.

In models with SES as mediators, differences were reduced the most between the groups, when all SES indicators where included in the models. However, in women, analyses that only included income and housing showed that employment contributed the most to the differences between the population groups. Another important aspect to keep in mind is the fact that employment status among immigrants might reflect a healthy worker effect and not a causal effect. In addition, income and employment might be inadequate indicators of SES among the elderly. In contrast, housing is most likely a better measurement of material wealth throughout the life-course. Education is stable and therefore it can be used over time, but was not included due to poor validity. However, a survey has reported a higher educational level among former Yugoslavians, compared with Turks and Pakistanis [23]. Therefore, it cannot be ruled out that former Yugoslavians' relatively low incidence of CVD and AMI is influenced by a high educational level. Moreover, the broadly defined categories of employment and housing conditions leaves a possibility that results might be biased due to residual confounding. This might also explain the lack of significant interaction terms between SES indicators and country of birth.

Cardiovascular disease in immigrants is likely to be influenced by factors previous to migration, the process of migration and post migration, as well as genetics might also play a certain role. Pre-migration factors might include lack of access to healthcare and poor socioeconomic status. The process of migration might be associated with stress, loss of network and discrimination in the new country. Stress is likely to trigger the progression of atherosclerotic processes in the arteries [24]. According to Hedlund et *al*., foreign-born individuals had an elevated risk of MI in the first year after immigration to Sweden, which might be associated with post migration stress [9]. Some studies have suggested that the prevalence of insulin resistance leading to diabetes and lipid abnormalities is higher among individuals identified as South Asians (Indians, Pakistanis and Bangladeshi) possibly due to a predisposition [25,26]. This fact might partially explain the increased incidence of CVD among Pakistanis in our study. Other studies have focused on country of birth-related differences in health behaviour, as an explanation for the increased CVD among immigrants. Major CVD risk factors such as diabetes, hypertension and smoking are overrepresented among some non-Western immigrants, compared with non-immigrants [27-30], which is also the case in a Danish survey of immigrants' health [31].

Duration of residence and acculturation in the new country might influence the risk of CVD morbidity among immigrants; however, the literature has shown contradictory findings in relation to the impact of duration of residence on CVD in immigrants [9,32,33]. In our study, we found that duration of residence had an impact on the incidence of CVD, although the applied categories for duration of residence were very broad. Among Pakistanis and Turks, we found a lower incidence of CVD among those with a shorter duration of residence. Acculturation may lead to improving health behaviours and access to medical care, but on the contrary, it can also promote unhealthy behaviour in the receiving country. The observed reverse pattern among former Yugoslavians might reflect that the disease pattern, with time, might become more similar to that of the receiving country. Another possible explanation for this finding is "the unhealthy re-migration effect", which has been described as the remigration of either ill, unhealthy or less successful migrants to their country of origin [3,34,35], leaving a healthier selection of individuals in the host country.

The main strength of this study is its registry-based design, including information on emigrations and deaths. The validity of CVD and AMI is high, as the National Patient Registry has nearly complete coverage [36,37]. Furthermore, the measurements of SES were based on individual data, included three different indicators and the fact that we were able to measure SES prior to follow-up strengthened the study. On the other hand the broad categories of SES leave a possibility of biased results and the omission of education data, due to poor validity, was a limitation. Our study has other noteworthy limitations. First, cases of AMI were limited among immigrants due to the groups' relatively small size. Secondly, deaths and morbidity related to CVD and AMI in the immigrant's country of birth during follow-up might underestimate the findings. Thirdly, the re-migration of less successful migrants before they become manifestly ill might also underestimate the findings. Fourthly, in case of a country of birth-related selection, when choosing between seeking healthcare and not seeking healthcare for CVD symptoms, the findings in our study might be biased. Studies have shown that perceived CVD symptoms and the acting (or not acting) on symptoms is likely to differ by ethnicity and country of birth [38-40]. Finally, cases of CVD not leading to hospital care are not included in this study; however, only if the selection between no-care, primary care and hospital care differs according to country of birth, the results might be biased. We would not expect such strong bias in case of AMI, but for other less acute CVD's there might be a tendency that immigrants more often go to the hospital directly, even if they are expected to be treated or referred by their general practitioner.

## Conclusions

The incidence of CVD and AMI varied according to country of birth; immigrants born in Pakistan and Turkey had an increased incidence of AMI and CVD, compared with native Danes. However, the incidence of CVD among immigrants from the former Yugoslavia did not differ notable from that of native Danes. The study highlights the importance of looking at each immigrant group individually instead of gathering them into one seemingly heterogeneous group. Differences between immigrants groups and native Danes were reduced when considering SES, but stratified analyses suggested that the relationship between some SES indicators and CVD incidence might differ by country of birth. These facts suggest that future studies should collect and test different SES indicators and conduct different analyses in the investigation of immigrants' health. Finally, the study suggests that country of birth is an important factor, when drawing attention to immigrant groups, where preventive efforts are needed in relation to CVD. The results indicate that more research is necessary to understand differences in incidence rates by country of birth, in particular, in terms of understanding how migration patterns affect incidence of CVD.

## Competing interests

The authors declare that they have no competing interests.

## Authors' contributions

NFH conceived the study, conducted the analysis of the data and had primary responsibility for drafting the manuscript. TJ, FBL, FD and AHA assisted with the study design and supervised the statistical analysis. SSN, FBL, FD, AHA and TJ participated in the interpretation of results and critically reviewed the manuscript. All authors read and approved the final manuscript.

## Pre-publication history

The pre-publication history for this paper can be accessed here:

http://www.biomedcentral.com/1471-2458/11/662/prepub
